# A hospital-based cancer registry in Luanda, Angola: the *Instituto Angolano de Controlo do Cancer* (IACC) Cancer registry

**DOI:** 10.1186/s13027-019-0249-2

**Published:** 2019-11-08

**Authors:** Fernando Miguel, Maria José Bento, Gonçalo Forjaz de Lacerda, Elisabete Weiderpass, Lúcio Lara Santos

**Affiliations:** 1 Angolan Institute of Cancer Control, Luanda, Angola; 2 Department of Epidemiology, Portuguese Institute of Oncology, Porto, Portugal; 3 Cancer Epidemiology Research Group, Portuguese Institute of Oncology, Porto, Portugal; 4 Division of Cancer Control and Population Sciences, National Cancer Institute, National Institutes of Health, Rockville, MD USA; 5 International Agency for Research on Cancer, Lyon, France; 6 Experimental Pathology and Therapeutics Group and Surgical Oncology Department, Portuguese Institute of Oncology, Rua Dr. António Bernardino de Almeida, PT–4200-072 Porto, Portugal; 7 ONCOCIR- Education and Care in Oncology in Lusophone African Countries, Porto, Portugal

**Keywords:** Angola, Luanda, Cancer registry

## Abstract

**Background:**

The Instituto Angolano de Controlo do Cancer (IACC) Cancer Registry in Luanda, Angola is the most ancient and organized hospital-based cancer registry in Angola and provides data on cancer cases treated in several hospital facilities in Luanda.

**Methods:**

Newly-diagnosed cancer cases (2012–2016) of IACC were collected. A total of 6638 malignant neoplasms were recorded. After excluding duplicates, missing data and non-melanoma skin cancers cases, a final number of 5609 cancer cases was considered valid for analysis.

**Results:**

From 5609 new cases, 2059 were males and 3550 females. Of all cases, 9.7% was in children below the age of 15 years. Most of the cases were residents from the Luanda district. The five most common cancers for all periods were breast (21.4%), cervix (16.8%), prostate (7.1%), non-Hodgkin lymphoma (4.5%) and Kaposi sarcoma (4.3%). For men, 19.3% of the cancers were prostate, 7.5% Kaposi sarcoma and 7.5% non-Hodgkin lymphoma. Cancers of the breast and cervix together accounted 60% of all cancers in females. Comparison of our data onto the 5 most frequent tumours, by sex, according to GLOBOCAN 2018 estimations for Angola, highlights the potential deviation from reality that estimates may have and reinforces the urgent need to build a truly population-based cancer registry in Luanda.

**Conclusion:**

To accomplish that task, it is mandatory to implement a more rigorous quality control program at the hospital-based cancer registry at IACC and to optimize the network of health institutions that actively working on and contributing to the cancer registry, in Luanda.

## Introduction

In Africa, infectious diseases are still a common and an important public health challenge; however, these disease patterns are changing, with a significant increase in the incidence rates of chronic diseases such as diabetes, hypertension, and cancer. Increased life expectancies and changing lifestyles, in addition to the high prevalence of specific infections are important contributing factors to the increased cancer burden [1].

Cancer registries are an important public health surveillance tool by providing a census of cancer cases and are a major source of critical information for the planning of cancer control measures such as prevention, early detection, treatment and care [2]. A February 2016 workshop held in Brazzaville, Republic of the Congo was co-organized by the International Atomic Energy Agency (IAEA), the World Health Organization’s Regional Office for Africa (WHO-AFRO), the International Agency for Research on Cancer (IARC), the African Cancer Registry Network (AFCRN) and experts of Republic of the Congo-Brazzaville and concluded that “collecting patients’ data in a comprehensive national registry is key to improve and effect cancer care in Africa” [3]. Unfortunately, most African countries, including Angola, do not have a population-based cancer registry [4], which limits the planning of tailored national and/or regional anti-cancer policies and calculation of cancer incidence and mortality rates and other important measures. In fact, for Angola cancer incidence and mortality rates were estimated by GLOBOCAN based on data from neighbouring countries [5] and not from Angola itself. In June 2012, an integrated mission of IAEA’s Programme of Action for Cancer Therapy (imPACT) to the Republic of Angola recommended to the Ministry of Health of Angola (MOH) that cancer registration in the country be improved, which included training staff in cancer registration methods.

**Fig. 1:**
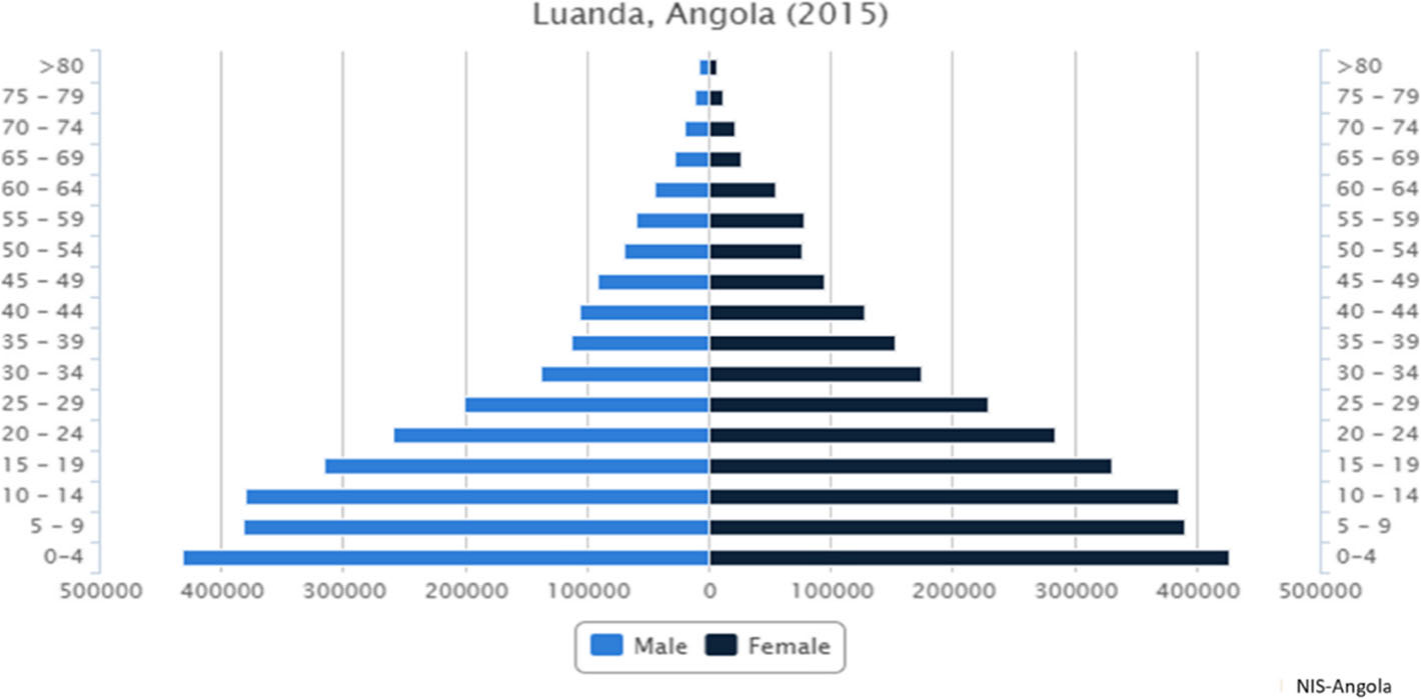
Luanda city population pyramid estimated for 2015. NIS – National Institute of Statistics

The Instituto Angolano de Controlo do Cancer (IACC) in Luanda (formerly the National Oncology Centre), is the oldest public centre for the treatment of cancer patients in Angola and hosts a hospital-based cancer registry. In 2015, Armando A et al. published the most common cancers in the IACC registry from 2007 to 2011, namely cervix cancer, breast and prostate cancer [6]. As the majority of cancer cases in Angola are treated in this facility, the IACC’s registry holds the potential to provide an estimate of the epidemiological profile of cancer in the Angolan population. Acknowledging this potential, in 2014 AFCRN experts recommended consolidating the existing IACC cancer registry and transforming it into a population-based cancer registry for the region of Luanda. Luanda is the largest city in Angola with 5,531,546 inhabitants in 2015 [7]. (Fig. 1).

The present study aims to describe the structure of the IACC’s hospital-based cancer registry (HBCR) including its challenges with data collection, reporting, and analysis in addition to providing an overview of the most frequent tumours, by age and gender in the registry. The potential for the registry to be used for estimating population-level cancer incidence rates for Angola and to propose priorities for activities to transform the IACC hospital-based cancer registry into a population-based cancer registry for Luanda are also discussed.

## Methods

### *Data sources* for the *registry*

The IACC hospital-based cancer registry provides the majority of the specialized cancer management services in Angola. The HBCR/IACC record for each cancer patient the following data: first name and surname, date of birth or approximate age, identification number, gender, marital status, ethnic origin, current residential address, place of birth, date of cancer diagnosis, histology/cytology number, tumour site and histology type, clinical stage of disease, basis of diagnosis, treatment given, vital status, and date and cause of death.

The registry employs passive methods of case finding and receives information from two semi-private institutions in Luanda, *Clínica Sagrada Esperança* and *Clínica Girassol,* which are involved in the management of cancer patients [6]. The HBCR/IACC also receives notifications from the *Américo Boavida Hospital*, *Josina Machel Hospital*, *Lucrécia Paim Maternity Hospital*, *and David Bernardino Pediatric Hospital* on a voluntary basis, as well as, information about cancer patients referred by the Health Minister for treatment abroad.

The primary data sources are the inpatient wards and oncology outpatient clinics of IACC. Additionally, records of specific cancer research studies are included. Information from the other public hospitals in Luanda, (*Prenda* Hospital, Central Military Hospital, *Augusto Ngangula* Maternity) is only included in the registry when the patient is transferred to the IACC for eventual treatment or multidisciplinary team discussion. Currently, there is no active case finding and minimal follow-up of cases performed by HBCR/IACC.

### Quality of the data

In Angola, the diagnosis of primary tumours is mainly obtained with the support of histology and cytology data, but clinical diagnoses reported by hospitals are also included. Death certificates are used less frequently as they are considered to be of poor quality.

### Data collection and storage

Each case report form is coded and entered into a Microsoft Excel spreadsheet created for the HBCR/IACC. Since 2014, the HBCR began using IARC’s CanReg5 to store, check, and validate the records. Each case receives a unique cancer registry identification number and the tumour site and morphology are coded according to the third edition of the International Classification of Diseases for Oncology [8, 9].

### Data analysis

We performed a descriptive analysis of the IACC HBCR data for the time period 2012–2016. Cases with missing or incomplete data were excluded from the analysis. In addition, this analysis included an evaluation of data consistency according to AFCRN recommendationsand results are provided in numbers and proportions [8].

Age-standardized (ASR) incidence rates for the Luanda region expressed per 100,000 person-years, considering only the cases diagnosed in Luanda, were computed by the direct method using the World Health Organization’s standard population as the reference [10]. All hypothesis tests were two-sided, and *P* values < 0.05 were considered statistically significant. Statistical analyses were performed using SPSS version 21 (New York, NY).

## Results

### Analysis of new reported cancer cases and method of diagnosis

From 2012 to 2016, 6638 new cancer cases were registered in the HBCR at IACC. Of these, 644 cases (10%) were considered duplicates and 200 cases (3%) were not assigned a diagnosis and excluded from the analyses. From 5794 cases, the proportion of cases which had a microscopically verification (histology or cytology) was 92.3, 6.7% had clinical diagnosis only, and 1.0% were registered through death certificates only. Non-melanoma skin cancers were diagnosed in 185 patients, and excluded from further analysis, which is the standard practice in cancer registry reports and allows for international comparisons. The final analyses were conducted on 5609 new cancer cases.

### Analysis of the distribution of reported cancers by age, site and gender

Of the 5609 cancer cases, the mean age at diagnosis was 45.4 years with a standard deviation (SD) of 19.5 years. Females accounted for 63.3% of the cancer cases in the HBCR. Age at diagnosis was not statistically significant between males and females (45.2 and 45.5, respectively, *p* = 0.65). The male/female ratio was 0.58.

For the time period studied, the five most common cancers were: breast (21.4%), cervix (16.8%), prostate (7.1%), non-Hodgkin lymphoma (4.5%) and Kaposi sarcoma (4.3%). For males, 19.3% of the cancers were prostate, 7.5% Kaposi sarcoma, and 7.5% non-Hodgkin lymphoma. Cancers of the breast and cervix together accounted for almost 60% of all cancers in females (Table 1).

**Table 1:** Distribution of the number of cancers by site and gender from the IACC hospital-based cancer registry, 2012–2016

	Total		Male		Female	
Cancer Site	No.	%	No.	%	No.	%
Lip and Oral Cavity	175	3.1	103	5.0	72	2.0
Nasopharynx	24	0.4	18	0.9	6	0.2
Oesophagus	132	2.4	95	4.6	37	1.0
Stomach	175	3.1	90	4.4	85	2.4
Colon and Rectum	155	2.8	94	4.6	61	1.7
Liver	141	2.5	85	4.1	56	1.6
Gallbladder	16	0.3	6	0.3	10	0.3
Pancreas	34	0.6	19	0.9	15	0.4
Nasal Cavity and Middle Ear	30	0.5	16	0.8	14	0.4
Larynx	76	1.4	65	3.2	11	0.3
Lung	99	1.8	69	3.4	30	0.8
Melanoma of Skin	56	1.0	21	1.0	35	1.0
Bone	68	1.2	36	1.7	32	0.9
Kaposi Sarcoma	241	4.3	154	7.5	87	2.5
Connective and Soft Tissue	182	3.2	96	4.7	86	2.4
Breast	1200	21.4	34	1.7	1166	32.8
Cervix Uteri	944	16.8	–	–	944	26.6
Corpus Uteri	74	1.3	–	–	74	2.1
Ovary	76	1.4	–	–	76	2.1
Prostate	397	7.1	397	19.3	–	–
Kidney	190	3.4	99	4.8	91	2.6
Bladder	76	1.4	33	1.6	43	1.2
Eye	181	3.2	83	4.0	98	2.8
Brain and CNS	41	0.7	15	0.7	26	0.7
Thyroid	37	0.7	13	0.6	24	0.7
Hodgkin Lymphoma	40	0.7	21	1.0	19	0.5
Non-Hodgkin Lymphoma	253	4.5	154	7.5	99	2.8
Multiple Myeloma	43	0.8	16	0.8	27	0.8
Leukaemia	135	2.4	76	3.7	59	1.7
Other and Unspecified Cancers	318	5.7	151	7.3	167	4.7
All cancers, excluding non-melanoma skin cancer	5609	100	2059	100	3550	100

Legend: *CNS* Central Nervous System

### Analysis of the distribution pattern of reported cancers by site and year of diagnosis

Between 2012 and 2016, the highest number of registered cases was 1232 new cases in 2013 (22% of all periods) and the lowest was in 2012 with 974 (17%) new cases in this quinquennium (Table 2, Additional file 1). Of the ten most common types of cancer, prostate and non-Hodgkin lymphoma, displayed a very different distribution pattern of the other common cancer types over the specified time period. The gender distribution was similar from 2012 to 2016.

**Table 2:** Distribution of the number of cancers by site and year of diagnosis from the IACC hospital-based cancer registry, 2012–2016. Distribution of all reported cases from 2012 to 2016 by gender is available in supplementary data (Additional file 1)

Cancer Site	2012	2013	2014	2015	2016	Total
Lip and Oral Cavity	17	35	39	43	41	175
Stomach	33	38	38	31	35	175
Kaposi Sarcoma	49	57	49	44	42	241
Connective and Soft Tissue	33	40	34	38	37	182
Breast	218	308	194	244	236	1200
Cervix Uteri	171	175	203	195	200	944
Prostate	109	88	41	92	67	397
Kidney	40	33	51	37	29	190
Eye	30	28	45	47	31	181
Non-Hodgkin Lymphoma	52	73	44	56	28	253
Other and Unspecified Cancers	222	357	386	359	347	1671
Total	974	1232	1124	1186	1093	5609

The mean age at diagnosis was 45.4 years old, SD ± 19.5 (45.2 for males and 45.5 for females). Breast and cervical cancers were the two most frequent cancer types each year. Other than breast and cervical cancers, there were variations in the relative frequency of the other cancer types, such as prostate cancer, non-Hodgkin lymphoma, Kaposi sarcoma, and cancers of the oral cavity, which were amongst the most commonly diagnosed.

### Analysis of the distribution pattern of reported cancers by place of residence

From all cancer cases, over 80 % (4607 cases) in the HBCR were residents from the Luanda district. There were no differences in the proportions of breast (Luanda residents - 33% vs people living in other areas of Angola- 32%) and cervix uteri (Luanda - 27% vs people living in other areas of Angola - 26%) cancers. However, there were a higher proportion of prostate cancer cases among patients living in urban Luanda (Luanda residents - 20% vs people living in other areas of Angola - 5%). On the contrary we found higher proportion of retinoblastoma (Luanda - 3% vs people living in other areas of Angola - 5%), kidney cancer (Luanda - 3% vs people living in other areas of Angola - 5%), and non-Hodgkin lymphoma (Luanda - 4% vs people living in other areas of Angola - 6%) diagnosed in residents living outside Luanda.

### Analysis of the distribution pattern of reported cancers in children (< 15 years old)

There were 543 new cancer cases (9.7%) reported over 2012–2016 among paediatric patients in the IACC HBCR aged less than 15 years. Of these 543, slightly more than half (55.6%) were male. Amongst this patient population, the most common cancer types were Wilms’ tumour, retinoblastoma, and non-Hodgkin lymphoma, corresponding to 64.4% of the total of cancers reported for this age group (Table 3).

**Table 3:** Distribution of the number and percentage of childhood cancers by site and gender from the IACC hospital-based cancer registry, 2012–2016

	Total		Male		Female	
Cancer Site	No	%	No	%	No	%
Connective and Soft Tissue	41	7.6	25	8.3	16	6.6
Wilms’ Tumour	141	26.0	71	23.5	70	29.0
Retinoblastoma	121	22.3	61	20.2	60	24.9
Non-Hodgkin Lymphoma	88	16.2	62	20.5	26	10.8
Leukaemia	47	8.7	27	8.9	20	8.3
Other and Unspecified Cancers	105	19.3	56	18.5	49	20.3
All cancers, excluding non-melanoma skin cancer	543	100.0	302	100.0	241	100.0

### Calculation of age-standardized incidence rates for the Luanda region

**Fig. 2:**
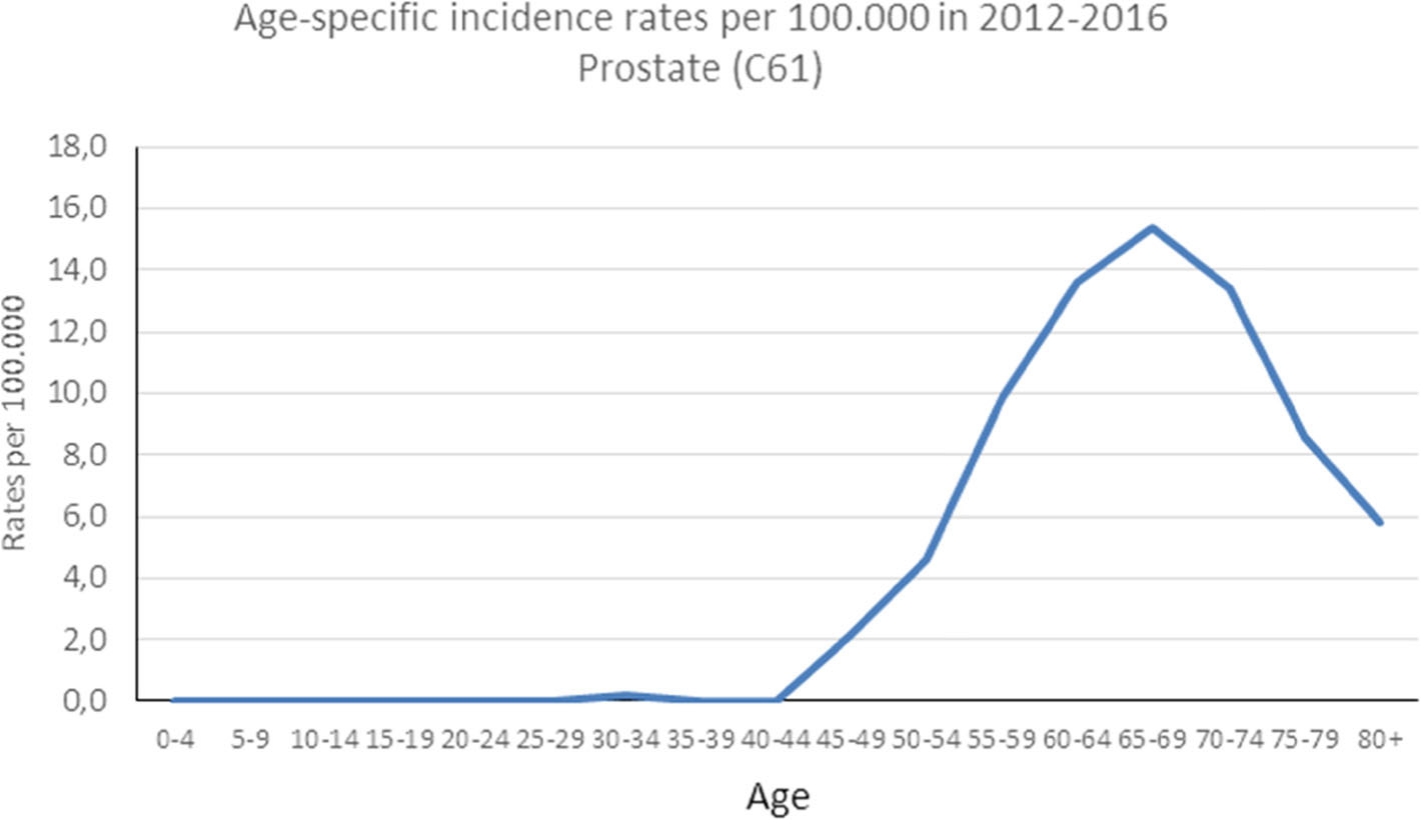
Age-specific incidence rates per 100,000 for prostate cancer from the IACC hospital-based cancer registry, 2012–2016

**Fig. 3:**
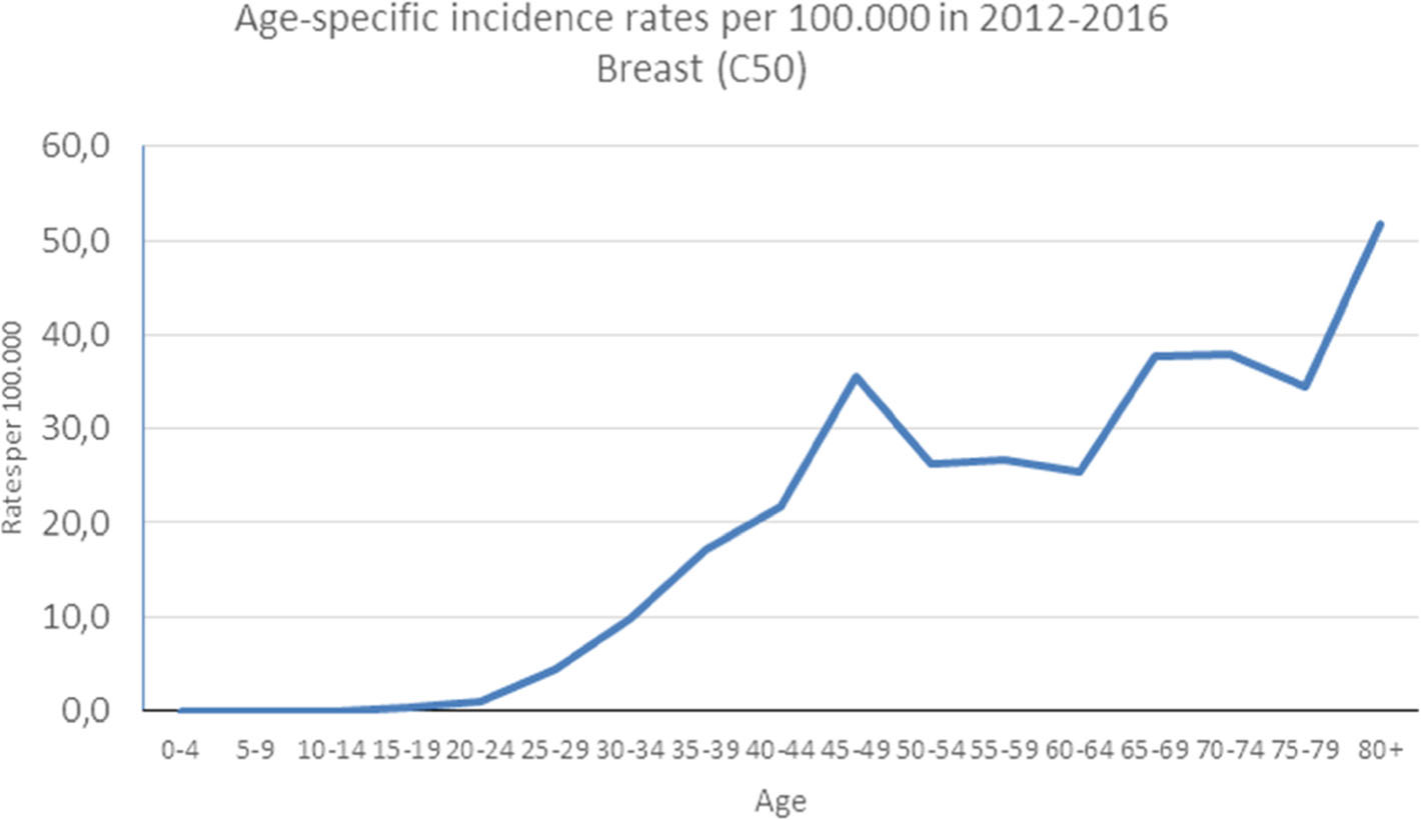
Age-specific incidence rates per 100,000 for breast cancer from the IACC hospital-based cancer registry, 2012–2016

**Fig. 4:**
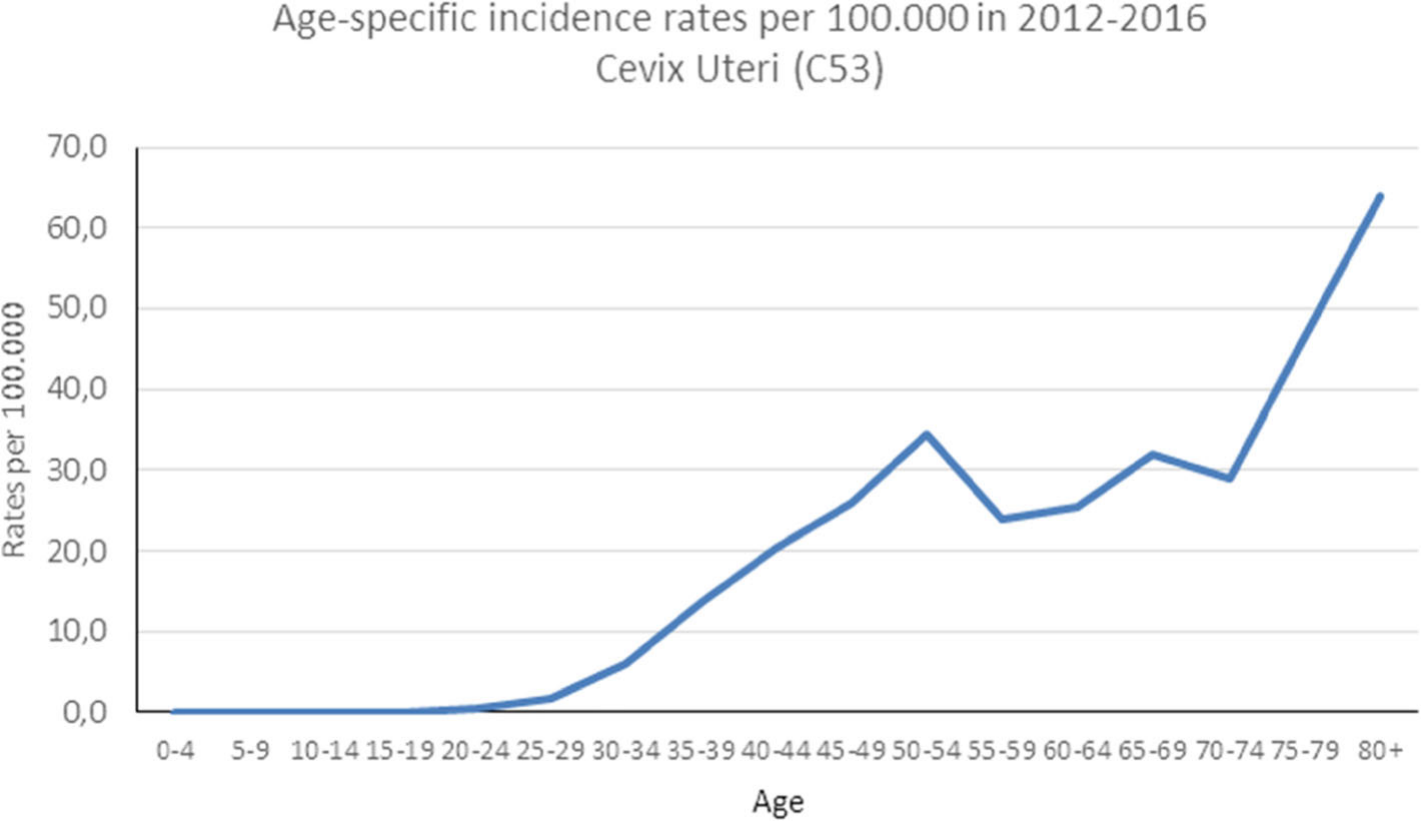
Age-specific incidence rates for cervix uteri cancer from the IACC hospital-based cancer registry, 2012–2016

The age-standardized incidence rates of the five most common cancer types for the Luanda region were calculated (Table 4). In males, the highest age-standardized incidence rate was prostate (7.9) followed by Kaposi sarcoma (1.8), lip and oral cavity (1.4), stomach (1.4) and non-Hodgkin lymphoma (1.22) at the lowest. In females, the highest age-standardized incidence rate was for breast (13.7) followed by cervix uteri (11.7), stomach (1.3), non-Hodgkin lymphoma (0.8) and Kaposi sarcoma (0.8) with the lowest rate. Figures 2, 3, and 4 show the age-specific rates for prostate, breast, and cervix uteri cancers, respectively.

**Table 4:** Top-five age-standardized incidence cancer rates per 100,000 person-years in the Luanda region, by gender from the IACC hospital-based cancer registry, 2012–2016

	Age-Standardized Rates per 100,000	
Rank	Male	Female
1	Prostate – 7.9	Breast – 13.7
2	Kaposi Sarcoma – 1.8	Cervix Uteri – 11.7
3	Lip and Oral Cavity – 1.4	Stomach – 1.3
4	Stomach – 1.4	Non-Hodgkin Lymphoma – 0.8
5	Non-Hodgkin Lymphoma – 1.2	Kaposi Sarcoma – 0.8

We compared our HBCR data to the five most common tumours, by gender according to IACR’s GLOBOCAN 2018 (Table 5). Data from Namibia, the Republic of the Congo-Brazzaville, and Zambia (all bordering Angola), were used to calculate the GLOBOCAN 2018 estimates [11]. There are differences between the most common tumours observed in the IACC HBCR and those published by GLOBOCAN.

**Table 5:** Estimated Top-five cancer types in 2018 (Angola, Congo, Zambia and Namibia) and Luanda Region (2012–2016)

Country	Luanda Region (2012–2016)		Angola^a^ GLOBOCAN 2018		R. Congo^b^ GLOBOCAN 2018		Zambia^c^ GLOBOCAN 2018		Namibia^d^ GLOBOCAN 2018	
	Men	Women	Men	Women	Men	Women	Men	Women	Men	Women
**1st**	**Prostate**	**Breast**	**Prostate**	Cervix U.	**Prostate**	**Breast**	**Prostate**	Cervix U.	**Prostate**	**Breast**
**2nd**	**Kaposi S.**	**Cervix U.**	Liver	Breast	Liver	**Cervix U.**	**Kaposi S.**	Breast	**Kaposi S.**	**Cervix U.**
**3th**	**Lip, Oral Cavity**	**Stomach**	Colorectal	Colorectal	Colorectal	Colorectal	NHL	Kaposi S.	**Lip, Oral Cavity**	Kaposi S.
**4th**	**Stomach**	**NHL**	NHL	Eye	**Stomach**	Ovary	Oesophagus	**NHL**	Colorectal	Colorectal
**5th**	**NHL**	**Kaposi S.**	Lung	Ovary	Lung	Liver	Colorectal	Colorectal	Lung	Ovary

*NHL* non-Hodgkin lymphoma; *Kaposi S*. Kaposi sarcoma; *Cervix U* Cervix uteri. ^a^Estimative according simple average of Congo, Zambia and Namibia rates. ^b^Rates from Brazzaville (2010–2014) applied to 2018 population; ^c^Rates from Lusaka (2011.-2015) applied to 2018 population; ^d^Rates from Namibia (2010–2014) 4 applied to 2018 population. Bold - cancer site equal across the different estimations and the Luanda region.

## Discussion

The cancer registries in most Sub-Saharan Africa countries are constrained in the collection of all cancer cases whether throughout the entire country or in specific regions. Therefore, accurate incidence rates are almost impossible to calculate due to lack of resources and organization to collect the needed data [12], which is the current situation in Angola. The best cancer registries occur in hospital units and where there is willingness to coordinate the collection and storage of these records centrally. However, creating cancer registries in hospitals, which was one of the recommendations of the AFCRN in 2014, has not been adequately implemented in Sub-Saharan Africa. This is a challenge in Angola due to several difficulties in expanding the current IACC hospital-based registry into a population-based registry.

### Characterization of HBCR/IACC

Currently, the IACC is insufficiently staffed to conduct active data collection in other hospitals, pathology laboratories, and health care facilities in Luanda. Moreover, information from death certificates is not available from General Register Offices and the existing death certificate information that is available is considered by the professionals interviewed during the AFCRN site visit to be of too poor quality to be useful for inclusion in a cancer registry. In this study, we identified several issues related to the quality of the information available in the IACC HBCR, such as missing data, duplicate case records, and data inconsistencies [13]. However, despite these limitations, the only data currently available in Angola is from the IACC hospital-based registry.

### Valuable information and limitations identified

Despite these data limitations available in the HBCR, our study is the largest, most complete, and the only study in which quality control of the data was conducted amongst cancer incidence studies conducted in Angola. In this study, missing and incomplete data were excluded from the analyses. In comparison to the data previously published by Armando and colleagues [6]. Regarding IACC HBCR for the time period 2007 to 2011, we verified that the global number of malignant tumours diagnosed per year increased. Cervical, breast, and prostate cancers are in the top three cancers ranked by incidence and accounted for a significant portion of the overall cancer burden in Angola. Interestingly, the number of cases with Kaposi’s sarcoma decreased slightly in comparison to the previous period of analysis [6].

The lower number of cases in 2016, as compared to the previous years can be explained in part, by the delay in the notification of new cancer cases and inconsistent data received from these other institutions that submit data to the IACC HBCR. However, the opening of other cancer care facilities and institutions and traveling for cancer treatment in Namibia and South Africa may also have contributed to the lower number of cases reported in Angola for this year [14].

The decreasing number of prostate cancer case records is likely due to underreporting. A private clinical unit dedicated to the treatment of urological cancer was established in Luanda; however, these cancer cases are not yet reported to the IACC HBCR and there was no urology clinic or specialized service for prostate cancer in the IACC during the studied period.

Regarding paediatric patients, Wilms’ Tumour, retinoblastoma and Non-Hodgkin’s Lymphoma were the most frequent tumours diagnosed at the IACC. The observed profile of paediatric cancers was similar to the prevalent tumours described recently by Wy and colleagues and by Stefan and colleagues when they analysed African paediatric cancer registries [15, 16].

In addition, we compared our data to the five most frequent tumours, by sex, according to GLOBOCAN 2018 for Angola. This comparison highlighted the potential deviation from the reality in many countries that estimates may have. A reliable study of cancer incidence rates, or time trends, integrating the limited existing data into mathematical models of incidence estimates may result in a closer and more realistic picture of cancer burden in Angola than the current models that only take into account estimates from neighbouring countries.

However, our results should not be considered in the GLOBOCAN estimates given that we observed too many fluctuations in numbers being diagnosed each year and other data limitations thereby suggesting a very large misclassification of some cancer cases at the IACC and other participating institutions.

### Proposal to optimize the IACC’s HBCR

Although the gold standard for cancer registration is population-based cancer registries, these are not always available in many countries [17, 18]. In these circumstances, HBCR may provide a starting point for determining cancer occurrence in a region or country. For example, Jedy-Agba and colleagues [19]. analyzed the role of hospital-based cancer registries in Nigeria and concluded that information provided by these registries is beneficial and could be used for the improvement of cancer care delivery systems in low and middle-income countries in the absence of population-based cancer registries. Thus, upgrading hospital-based cancer registries to population-based cancer registries in these regions is of paramount importance and should be a priority of a government [19].

As a result, dedicated oncology clinical units are under development in the most important general hospitals in Luanda and these units are comprised of a medical oncologist and a dedicated nurse. Tumour registrars, who will be part of the hospital registry service, are also included. Thus, a registry office for cancer cases in the main hospitals in Luanda was established and will serve as the primary source of information for the future population-based Luanda Cancer Registry. In Angola, despite our efforts in creating and maintaining the hospital-based cancer registry at the IACC, the true total burden of cancer, as well as, the details of cancer types, remains largely unknown.

It is strategic that cancer became a notifiable disease under Angolan law for both the public and private sectors. All cases of cancer diagnosed in Luanda hospitals, even without microscopic confirmation, are to be reported to population-based cancer registry, as proposed by the AFCRN [8, 20, 21]. The location of this cancer registry is (or should be), according to the Angolan law, the IACC [6]. All death certificates that mention cancer under the local health authority’s control must notify to the cancer registry. The newly-diagnosed cancer cases in the capital city should be actively collected from all hospitals, clinics, and diagnostic facilities. Additionally, the IACC hospital-based cancer registry quality control program should be instituted and followed by all hospitals where cancers are diagnosed and treated in Luanda (Table 6).

**Table 6:** IACC hospital-based cancer registry quality control program

Standards	Quality control activities	Place/support
Capacity building	Appropriate training of tumor registrars and research assistants	Portuguese Institute of Cancer, Porto, Portugal and Beira Cancer registry, Mozambique
Capacity building	Additional support provided by the Portuguese Institute of Cancer, Porto, Portugal cancer registry staff.	Portuguese Institute of Cancer, Porto, Portugal by teleconference
Internal organization	Using the Standard Procedure Manual for Population-Based Cancer Registry in sub Saharan Africa.	https://afcrn.org/index.php/resources2/53-standard-procedure-manual (20)
Internal organization	Using the AFCRN Data Collection Form	Portuguese translation of AFCRN Data Collection Form https://afcrn.org/index.php/resources2/56-data-collection-form (8)
Improving the quality of cancer registry	Comparing hospital medical record and tumor registry data	IACC
Improving the quality of cancer registry	Reviewing by research assistant to check accuracy of gender, age, histologic and morphologic diagnosis of the patients according to ICD-O 3,	IACC
Improving the quality of cancer registry	Crosschecks by research assistant to increase the consistency of the database	IACC
Improving the quality of cancer registry	Revise and record of inaccurate data	IACC
Improving the quality of cancer registry	Validation of data by using quality control programs/tools of International Agency for Research on Cancer (IARC) for avoiding duplication and any unlikely combination of age, sex, site and morphology and other factors in the database (CanReg 5).	IACC
Improve the cancer registry network in order to start an Population-based cancer registry of Luanda	Appropriate training of tumor registrars of other hospitals, in particular: Américo Boavida Hospital, Josina Machel Hospital, Lucrécia Paim Maternity Hospital, David Bernardino Pediatric Hospital, Prenda Hospital, Central Military Hospital and Augusto Ngangula Maternity	IACC and Portuguese Institute of Cancer, Porto, Portugal
workshop on the population-based cancer registry of Luanda	All hospitals in Luanda	IACC and Ministry of Health

## Conclusions

The IACC hospital-based cancer registry provides data on cancer cases treated in several hospital facilities in Luanda. However, this data cannot be considered a reflection of the situation in Luanda (nor Angola) as a whole, as data are not truly a population-based registry. Therefore, in order to overcome the IACC cancer registry limitations identified and to build a population-based cancer registry in Luanda, it is mandatory to implement a rigorous quality control program and to optimize the network of health institutions that actively working on and contributing to the cancer registry, in Luanda.

## Supplementary information

**Additional file 1.** Distribution of all reported cases from 2012 to 2016 by gender (supplementary data)

## Data Availability

The datasets used and/or analysed during the current study are available on IACC cancer registry in Luanda, Angola, a copy is also available at Epidemiology Department IPO-Porto. These data is available on justified request to the corresponding author. However, the crucial data generated or analysed during this study are included in this published article at the supplementary information table.
