# Supplementary material for: A hospital-based cancer registry in Luanda, Angola: the Instituto Angolano de Controlo do Cancer (IACC) Cancer registry
Source: Infect Agent Cancer. 2019 Nov 8;14:35. doi: 10.1186/s13027-019-0249-2 (PMC6839121; doi:10.1186/s13027-019-0249-2)
Supplement: Supplementary file 1 — Additional file 1. Distribution of all reported cases from 2012 to 2016 by gender (supplementary data) [file 13027_2019_249_MOESM1_ESM.docx]

|  | **Male** | | | | | | **Female** | | | | | | **Total** |
| --- | --- | --- | --- | --- | --- | --- | --- | --- | --- | --- | --- | --- | --- |
| **Cancer site** | **2012** | **2013** | **2014** | **2015** | **2016** | **Total** | **2012** | **2013** | **2014** | **2015** | **2016** | **Total** |  |
| **Lip, oral cavity** | 9 | 18 | 23 | 24 | 29 | 103 | 8 | 17 | 16 | 19 | 12 | 72 | 175 |
| **Nasopharynx** | 3 | 4 | 5 | 1 | 5 | 18 | 3 |  | 2 |  | 1 | 6 | 24 |
| **Oesophagus** | 12 | 14 | 20 | 25 | 24 | 95 | 8 | 8 | 11 | 7 | 3 | 37 | 132 |
| **Stomach** | 20 | 17 | 18 | 18 | 17 | 90 | 13 | 21 | 20 | 13 | 18 | 85 | 175 |
| **Colorectum** | 15 | 14 | 10 | 26 | 29 | 94 | 15 | 12 | 15 | 11 | 8 | 61 | 155 |
| **Liver** | 4 | 19 | 17 | 20 | 25 | 85 | 4 | 14 | 12 | 13 | 13 | 56 | 141 |
| **Gallbladder** |  | 1 |  | 3 | 2 | 6 |  | 2 | 2 | 2 | 4 | 10 | 16 |
| **Pancreas** | 1 | 5 | 5 | 5 | 3 | 19 | 1 | 2 | 4 | 3 | 5 | 15 | 34 |
| **Nasal Cavity and Middle Ear** | 3 | 2 | 3 | 2 | 6 | 16 | 3 | 3 | 4 | 4 |  | 14 | 30 |
| **Larynx** | 10 | 12 | 18 | 15 | 10 | 65 | 1 | 1 | 2 | 6 | 1 | 11 | 76 |
| **Lung** | 9 | 18 | 17 | 15 | 10 | 69 | 6 | 4 | 4 | 7 | 9 | 30 | 99 |
| **Melanoma of Skin** | 2 | 6 | 4 | 4 | 5 | 21 | 3 | 13 | 7 | 8 | 4 | 35 | 56 |
| **Bone** | 4 | 8 | 8 | 9 | 7 | 36 | 4 | 8 | 11 | 5 | 4 | 32 | 68 |
| **Kaposi Sarcoma** | 33 | 36 | 33 | 23 | 29 | 154 | 16 | 21 | 16 | 21 | 13 | 87 | 241 |
| **Connective and Soft Tissue** | 16 | 26 | 17 | 19 | 18 | 96 | 17 | 14 | 17 | 19 | 19 | 86 | 182 |
| **Breast** | 9 | 11 | 4 | 5 | 5 | 34 | 209 | 297 | 190 | 239 | 231 | 1166 | 1200 |
| **Cervix Uteri** |  |  |  |  |  |  | 171 | 175 | 203 | 195 | 200 | 944 | 944 |
| **Corpus Uteri** |  |  |  |  |  |  | 8 | 15 | 16 | 19 | 16 | 74 | 74 |
| **Ovary** |  |  |  |  |  |  | 12 | 23 | 18 | 14 | 9 | 76 | 76 |
| **Prostate** | 109 | 88 | 41 | 92 | 67 | 397 |  |  |  |  |  |  | 397 |
| **Kidney** | 18 | 23 | 29 | 13 | 16 | 99 | 22 | 10 | 22 | 24 | 13 | 91 | 190 |
| **Bladder** | 5 | 7 | 5 | 3 | 13 | 33 | 4 | 9 | 8 | 11 | 11 | 43 | 76 |
| **Eye** | 14 | 15 | 18 | 23 | 13 | 83 | 16 | 13 | 27 | 24 | 18 | 98 | 181 |
| **Brain and CNS** | 1 | 1 | 5 | 4 | 4 | 15 | 3 | 8 | 6 | 6 | 3 | 26 | 41 |
| **Thyroid** | 3 | 2 | 2 | 3 | 3 | 13 | 7 | 3 | 7 | 5 | 2 | 24 | 37 |
| **Hodgkin lymphoma** |  | 1 | 5 | 11 | 4 | 21 | 1 |  | 3 | 11 | 4 | 19 | 40 |
| **Non-Hodgkin lymphoma** | 26 | 46 | 29 | 34 | 19 | 154 | 26 | 27 | 15 | 22 | 9 | 99 | 253 |
| **Multiple myeloma** | 4 | 3 | 7 | 2 |  | 16 | 5 | 7 | 6 | 3 | 6 | 27 | 43 |
| **Leukaemia** | 9 | 15 | 18 | 13 | 21 | 76 | 9 | 14 | 13 | 9 | 14 | 59 | 135 |
| **Other cancers** | 16 | 37 | 38 | 29 | 31 | 151 | 24 | 42 | 48 | 25 | 28 | 167 | 318 |
| **All cancers, excluding non-melanoma skin cancer** | **355** | **449** | **399** | **441** | **415** | **2059** | **619** | **783** | **725** | **745** | **678** | **3550** | **5609** |

Distribution of all reported cases from 2012 to 2016 by gender (supplementary data)
